# Image Classification of Human Carcinoma Cells Using Complex Wavelet-Based Covariance Descriptors

**DOI:** 10.1371/journal.pone.0052807

**Published:** 2013-01-16

**Authors:** Furkan Keskin, Alexander Suhre, Kivanc Kose, Tulin Ersahin, A. Enis Cetin, Rengul Cetin-Atalay

**Affiliations:** 1 Electrical and Electronics Engineering Department, Bilkent University, Ankara, Turkey; 2 Department of Molecular Biology and Genetics, Bilkent University, Ankara, Turkey; Rice University, United States of America

## Abstract

Cancer cell lines are widely used for research purposes in laboratories all over the world. Computer-assisted classification of cancer cells can alleviate the burden of manual labeling and help cancer research. In this paper, we present a novel computerized method for cancer cell line image classification. The aim is to automatically classify 14 different classes of cell lines including 7 classes of breast and 7 classes of liver cancer cells. Microscopic images containing irregular carcinoma cell patterns are represented by subwindows which correspond to foreground pixels. For each subwindow, a covariance descriptor utilizing the dual-tree complex wavelet transform (DT-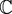
WT) coefficients and several morphological attributes are computed. Directionally selective DT-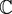
WT feature parameters are preferred primarily because of their ability to characterize edges at multiple orientations which is the characteristic feature of carcinoma cell line images. A Support Vector Machine (SVM) classifier with radial basis function (RBF) kernel is employed for final classification. Over a dataset of 840 images, we achieve an accuracy above 98%, which outperforms the classical covariance-based methods. The proposed system can be used as a reliable decision maker for laboratory studies. Our tool provides an automated, time- and cost-efficient analysis of cancer cell morphology to classify different cancer cell lines using image-processing techniques, which can be used as an alternative to the costly short tandem repeat (STR) analysis. The data set used in this manuscript is available as supplementary material through http://signal.ee.bilkent.edu.tr/cancerCellLineClassificationSampleImages.html.

## Introduction

Automatic classification of biomedical images is an emerging field, despite the fact that there is a long history of image recognition techniques [1]. Automated classification of carcinoma cells through morphological analysis will greatly improve and speed up cancer research conducted using established cancer cell lines as in vitro models. Distinct morphologies of different types and even sub-types of cancer cells reflect, at least in part, the underlying biochemical differences, i.e., gene expression profiles. Moreover, the morphology of cancer cells can infer invasivenes of tumor cell and hence the metastatic capability. The change in morphologies upon treatment with agents that induce cellular responses such as cell death or cell growth arrest [2]. Table 1 shows a summary of the different morphologies for the cancer cell lines in the dataset. In addition, an automated morphological classification of cancer cells will enable the correct detection and labelling of different cell lines. In molecular biology studies, experimenters deal with a large number of specimens whose identity have to be checked recurringly during different stages of the experiment. Therefore, predicting labels of cancer cell lines in a fast and accurate manner via a pattern classification approach will greatly enhance biologists’ ability to identify different types of cell lines without the need to scrutinize each and every microscopic image one by one. Although cell lines are being used widely as in vitro models in cancer research and drug development, mislabeling cell lines or failure to recognize any contamination may lead to misleading results. Short tandem repeat (STR) analysis is being used as a standard for the authentication of human cell lines. However, this process takes a long time and has to be carried out by an expert. Automated analysis, on the other hand, will provide the scientists a fast and easy-to-use tool that they can use in their own laboratories to verify their cell lines.

**Table 1:** Morphology of cancer cell lines used in this study.

	Morphology			Cancer Type		
Cell Line	Shape	Shape	Growth properties	Source	Classification	Disease
BT-20	epithelioid	stellate	adherent	mammary gland breast	Basal A	Adenocarcinoma
CAMA-1	epithelioid	grape-like	adherent	mammary gland breast	Luminal	Adenocarcinoma
MDA-MB-157	epithelioid	stellate	adherent	mammary gland breast	Basal B	Medullary carcinoma
MDA-MB-361	epithelioid	grape-like	adherent	mammary gland breast	Luminal	Metastatic adenocarcinoma
MDA-MB-453	epithelioid	grape-like	adherent	mammary gland breast	Luminal	Metastatic carcinoma
MDA-MB-468	epithelioid	grape-like	adherent	mammary gland breast	Basal A	Metastatic adenocarcinoma
T47D	epithelioid	mass	adherent	mammary gland breast	Luminal	Invasive ductal carcinoma
FOCUS	fibroblastoid	polygonal tospindle-shaped	adherent	liver	poorly differentiated	Hepatocellular carcinoma
Hep40	epithelioid	polygonal	adherent	liver	well differentiated	Hepatocellular carcinoma
HepG2	epithelioid	polygonal, grow as clusters	adherent	liver	well differntiated	Hepatocellular carcinoma
Huh7	epithelioid	polygonal	adherent	liver	well differentiated	Hepatocellular carcinoma
Mahlavu	fibroblastoid	polygonal to	adherent	liver	poorly	Hepatocellular
		spindle-shaped			differentiated	carcinoma
PLC	epithelioid	polygonal	adherent	liver	well differntiated	Hepatocellular carcinoma
SkHep1	fibroblastoid	polygonal tospindle-shaped	adherent	liver	poorly differentiated	Hepatocellular carcinoma

Modelling of cell morphology has been studied by several groups, for example for fission yeast in [3] and for e. coli bacteria in [4]. In the fission yeast case, differential expression of protein affects the cell size and, therefore, cell fate, while in the e. coli case, the topological organization is analyzed with respect to the underlying signaling network. To the best of our knowledge there have been no studies that have used morphology of different human cancer cell lines for classification.

Feature parameters are computed using the dual-tree complex wavelet transform (DT-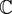
WT). In addition, directional difference scores and covariance descriptors are deployed in support vector machines (SVM) for analysis and classification of carcinoma cell line images. Detailed descriptions of these methods can be found in the feature extraction and classification sections; below we perform a literature search on how these techniques are applied in the medical domain. DT-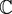
WT is a recently developed image decomposition method that possesses orientation selectivity and shift invariance properties lacking in the classical discrete wavelet transform. In the biomedical image analysis literature, DT-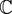
WT is used to predict the histological diagnosis of colorectal lesions in colonoscopy images by employing a probabilistic framework where a joint statistical model for complex wavelet coefficient magnitudes is proposed [5]. In [6], authors model the marginal distributions of DT-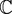
WT coefficient magnitudes by Rayleigh and Weibull probability density functions to classify the zoom-endoscopy images for colorectal cancer diagnosis. In [7], MR images of human brain and wrist are classified using textural features extracted via DT-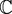
WT decomposition. Directional difference scores are first introduced in this article and applied to our classification problem. Normalized versions of covariance descriptor, which is a matrix-form feature describing an image region are used. In the medical domain, covariance descriptors are utilized for classification of colonic polyps in CT colonography images [8]. Our study is one of the first studies to apply the covariance descriptors to medical image analysis domain. SVM is a well-known machine learning algorithm that learns the decision boundaries between classes using separating hyperplanes. SVM is used in [9] for automated prostate cancer grading on histology images. In [10], a segmentation framework for cell microscopic images is proposed that adopts segmentation-by-classification approach and uses SVM for pixel classification. In [11], computer-aided classification of renal cell carcinoma subtypes is performed by using SVM. A fully automated system is presented for human cell phenotype monitoring in [12] and subcellular phenotypes on human cell arrays are automatically classified via SVM.

In this study, discrimination of 14 classes of biomedical images is achieved, which are all images of cancer cell lines. The dataset at hand consists of two major types of cancer cell lines, namely breast cancer and liver cancer (hepatocellular carcinoma) with 7 sub-classes, respectively. The dataset consists of 840 images, i.e., 60 per sub-class. Our approach aims to carry out the automated analysis by extracting a feature vector from the images. These feature parameters reflect the large morphological diversity of the images. Notice, however, that our software learns the specific covariances of these features from the training set, so the model for each image class is not rigid and therefore allows for larger variation in the image data, while maintaining its high effectivity.

This paper is organized as follows: We first present the experimental results and and then offer a brief discussion. In the Materials section, the used cell cultures are described. In the feature extraction section steps are described comprising image decomposition method by the dual-tree complex wavelet transform (DT-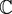
WT), directional difference score computation and covariance matrix construction. In the classification section, SVM based covariance matrix classification algorithm is explained along with the foreground-background segmentation by EM algorithm and random subwindow selection.

## Results

**Figure 1:**
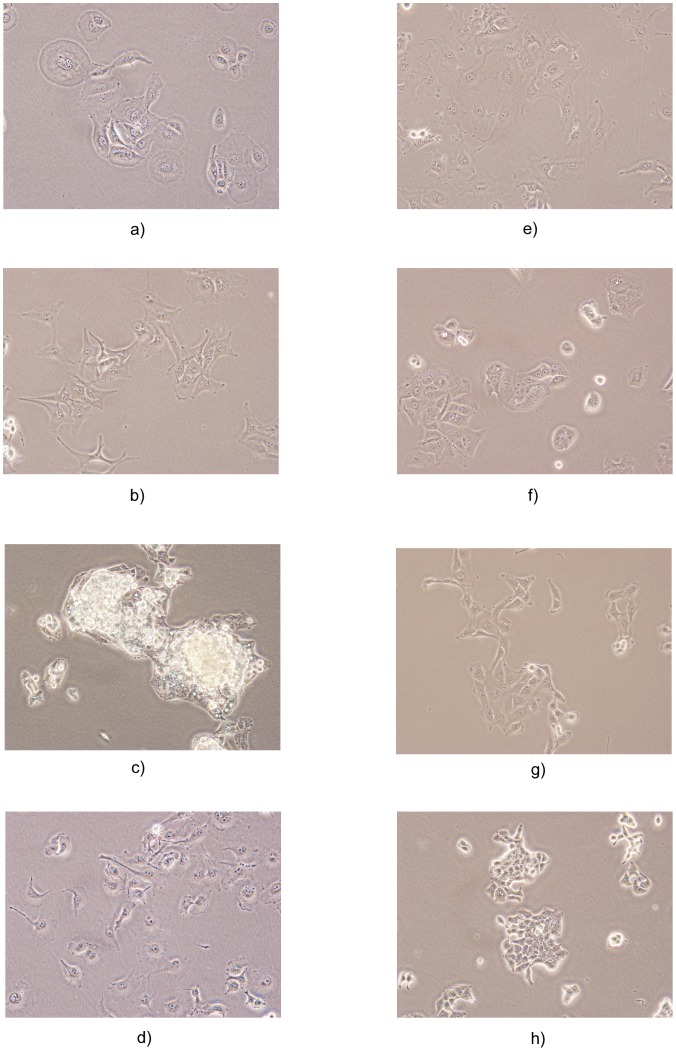
Sample images from different cancer cell line classes. a) BT-20, b) Focus, c) HepG2, d) MDA-MB-157, e) MV, f) PLC, g) SkHep1, h) T47D.

The dataset used in this study consists of 280 microscopic human carcinoma cell line images with each of the 14 classes having 20 images. Images in the dataset were acquired at 10×, 20× and 40× magnification. The size of each image was 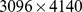 pixels. 7 classes belonged to breast cancer cell lines and the other classes belonged to liver cancer. Each cell type has a specific phenotype in terms of nuclei (spherical vs. ovoid), nucleoli (prominent vs. hardly noticeable), size (large vs. small) and shape (round vs. cell pods) [1]. The names of the cancer cell lines used in our study are shown in Table 2 and example images of all 14 classes are shown in Figure 1. Aggressive cancer cells with metastatic properties switch from an epithelial-like (epithelioid) morphology to a spindle-shaped fibroblast-like (fibroblastoid) morphology during epithelial-mesenchymal transition (EMT), which is an indication of the invasiveness and metastatic capability of cancer cells. While epithelioid cells have polygonal shape with regular dimensions and sharp boundaries, fibroblastoid cells have elongated shapes and are bipolar or multipolar.

**Table 2:** Names of cancer cell lines used in this study.

Breast cancercell line	Liver cancer cell line
BT-20	FOCUS
CAMA-1	Hep40
MDA-MB-157	HepG2
MDA-MB-361	Huh7
MDA-MB-453	Mahlavu
MDA-MB-468	PLC
T47D	SkHep1

**Figure 2:**
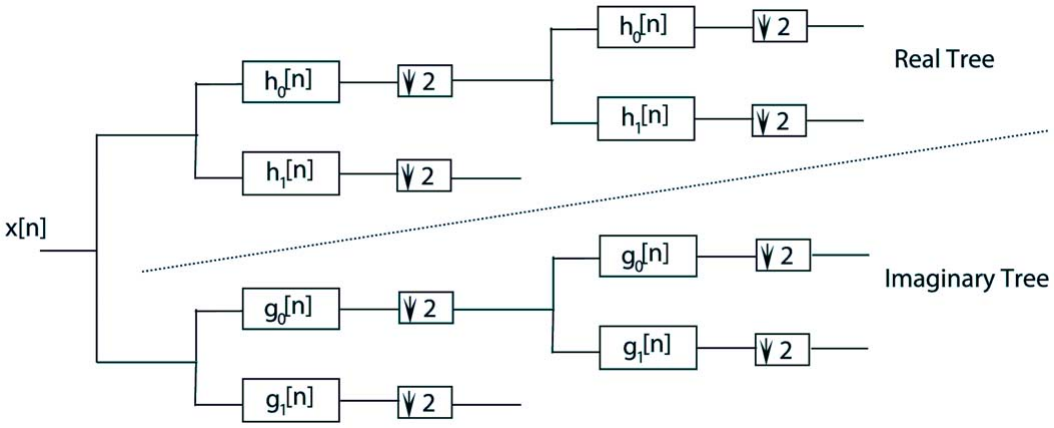
Examples of misclassified images (20×). Misclassified images are shown in the first column. Examples from their true cell line are given in the second column. Images in the third column show examples of the cell line that the images got misclassified into.

We adopt a 20-fold cross-validation strategy for the experiments. The dataset is divided into 20 disjoint subsets and each subset consisting of 14 images is used exactly once as the test set. For 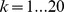, the 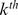 subset is formed by taking the 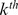 indexed image of each class. We run 20 experiments, choosing each image as the test image only once for each class, and obtain the average image classification accuracy over 20 runs. The number of selected random subwindows is taken to be 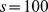. We perform the above experiment for both covariance and normalised covariance matrices, and for four different mapping functions in (10)-(13). SVM RBF kernel parameters are chosen as 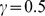 and 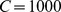. Experimental results are shown in Tables 3 for 10×, Table 4 for 20× and Table 5 for 40×. These tables show that normalised covariance matrix-based method outperforms the covariance method for all mapping functions, achieving an accuracy above 98%. Complex wavelet and directional difference features based classification methods (10)-(12) have higher accuracies than the classical covariance method in (13). Example images that were incorrectly classified are shown in Figure 2.

**Table 3:** Average classification accuracies (in %) of 10× carcinoma cell line images over 20 runs using SVM with RBF kernel.

Feature mapping function	Covariance -based classification	Normalised Covariance -basedclassification
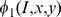	96.8	97.5
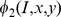	96.8	98.6
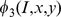	96.4	97.1
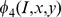	77.5	86.1

**Table 4:** Average classification accuracies (in %) of 20× carcinoma cell line images over 20 runs using SVM with RBF kernel.

Feature mapping function	Covariance -based classification	Normalised Covariance -basedclassification
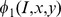	97.5	99.3
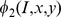	96.8	98.6
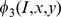	97.9	99.3
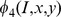	77.9	85.7

**Table 5:** Average classification accuracies (in %) of 40× carcinoma cell line images over 20 runs using SVM with RBF kernel.

Feature mapping function	Covariance -based classification	Normalised Covariance -basedclassification
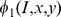	89.3	95.7
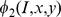	90.0	96.4
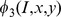	92.5	96.8
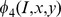	63.2	85.0

For comparison, similar experiments were carried out with scale-invariant feature transform (SIFT) [13] features. Table 6 shows the performance of those features. While the accuracy for discriminating between two cancer cell lines is 100%, the SVM classifier (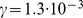 and 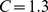) performs more poorly with each added cancer cell line. Furthermore, we investigated the effect of only using the diagonal of the normalised covariance matrix from Equation 7, i.e., the variance values of the features, as input for the SVM. Results can be seen in Table 7. The accuracy rates drop by approximately 10%. Therefore, using the covariances of the features is vital for a good performance of the system. It is clearly demonstrated via our experiments that image classification accuracy can be enhanced by exploiting the directional information through the use of DT-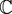
WT features and directional scores obtained by median, max and mean functions.

**Table 6:** Classification accuracies for SIFT features.

Number of cell lines	Classification accuracy in %
2	100.00
3	80.00
4	66.25
5	60.00
6	51.67
7	56.43
8	47.50
9	42.22
10	38.50
11	35.91
12	35.00
13	34.23
14	36.07

**Table 7:** Classification accuracies for variance values only.

Magnification	Classification accuracy in %
10×	84.60
20×	84.60
40×	80.00

## Discussion

The proposed automated system for human breast and liver cancer cell line images can aid the biologist as a second reader and avoid the need for costly and time-consuming biochemical tests. The dual-tree complex wavelet transform and region covariance based computational framework is successfully applied to classify the cancer cell line images. We adopt a covariance-based approach by exploiting pixel-level attributes to construct local region descriptors encoding covariances of several attributes inside a region of interest. Pixel attributes are extracted using directional difference scores and the DT-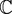
WT. Since background regions occur frequently in a cancer cell line image, we randomly sample subwindows from the foreground image regions after foreground-background segmentation and each microscopic image is represented by correlation matrices of certain number of subwindows sampled randomly from the whole image. Finally, an SVM classifier with RBF kernel is trained to learn the class boundaries.

Figure 2 juxtaposes example images of cell line A that gets misclassified as cell line B, with examples of both cell lines A and B. All images were recorded at 20×. The three cell lines shown in the figure that get misclassified are MDA-MB-468, Mahlavu and SKHep1. Some MDA-MB-468 images get misclassified as MDA-MB-361. Both are breast-cancer cell lines. From Figure 2, one understands that both images have layers, i.e., they have a 3-D structure, indicated by the white areas around the cell. This may be the reason why they get confused with one another. The liver cancer cell lines Mahlavu and SkHep1 are both misclassified as FOCUS, which is also a liver cancer cell-line. In the Mahlavu case, the image that gets misclassified shows several structures of significant length but short width, informally called “pods”. The FOCUS cell line has similar properties but, Mahlavu generally doesn't. Also, the misclassified image in the figure shows less informative morphological properties, other than most Mahlavu images. In the case of SkHep1, the example image shows a sparser structure than most SkHep1 images. In the second column of the figure there are two different example images from the FOCUS cell line in order to demonstrate its varying pod morphology bearing poor differntiation. In addition, this preliminary observation indicates that when the cell lines are poorly differentiated (as in FOCUS, Mahlavu and SkHep1), their morphology may vary, hence they are more prone to be misclassified [14]. This observation can be further investigated in the future with a larger dataset specific to these kind of undifferntiated cell lines.

We demonstrate that automatic classification of microscopic carcinoma cell line images can be reliably performed using DT-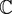
WT and correlation descriptors. Covariance descriptors are computed for features extracted from 2-D DT-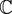
WT subbands and directional difference scores. Promising classification results were obtained by our experiments, which reveal the ability of the proposed features to characterize breast and liver carcinoma cell line textures.

## Materials and Methods

### 1 Cell Culture

The six hepatocellular carcinoma, one hepatoblastoma and seven breast cancer cell lines were obtained from the following sources: FOCUS ([15]), Hep40 ([16]), Huh7 (JCRB JCRB0403), Mahlavu ([17]), PLC (ATCC CRL-8024), SkHep1 (ATCC HTB-52), HepG2 (ATCC HB-8065), BT-20 (ATCC HTB-19), CAMA-1 (ATCC HTB-21), MDA-MB-157 (ATCC HTB-24), MDA-MB-361 (ATCC HTB-27), MDA-MB-453 (ATCC HTB-131), MDA-MB-468 (ATCC HTB-132), T47D (ATCC HTB-133). The cell lines were seeded into dishes with 20% confluency and grown at 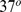
C under 5% CO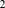 in standard Dulbecco’s modified Eagle’s medium (DMEM) supplemented with 10% FBS, 1% Non-Essential Aminoacid and 1% penicillin/streptomycin (GIBCO Invitrogen) up to 70% confluency. The authentication of the cell lines was regularly checked by STR profiling. Pictures were taken with Olympus CKX41 inverted microscope using Olympus DP72 camera with 20X objective.

### 2 Feature Extraction

#### 2.1 Dual-Tree complex wavelet transform

**Figure 3:**
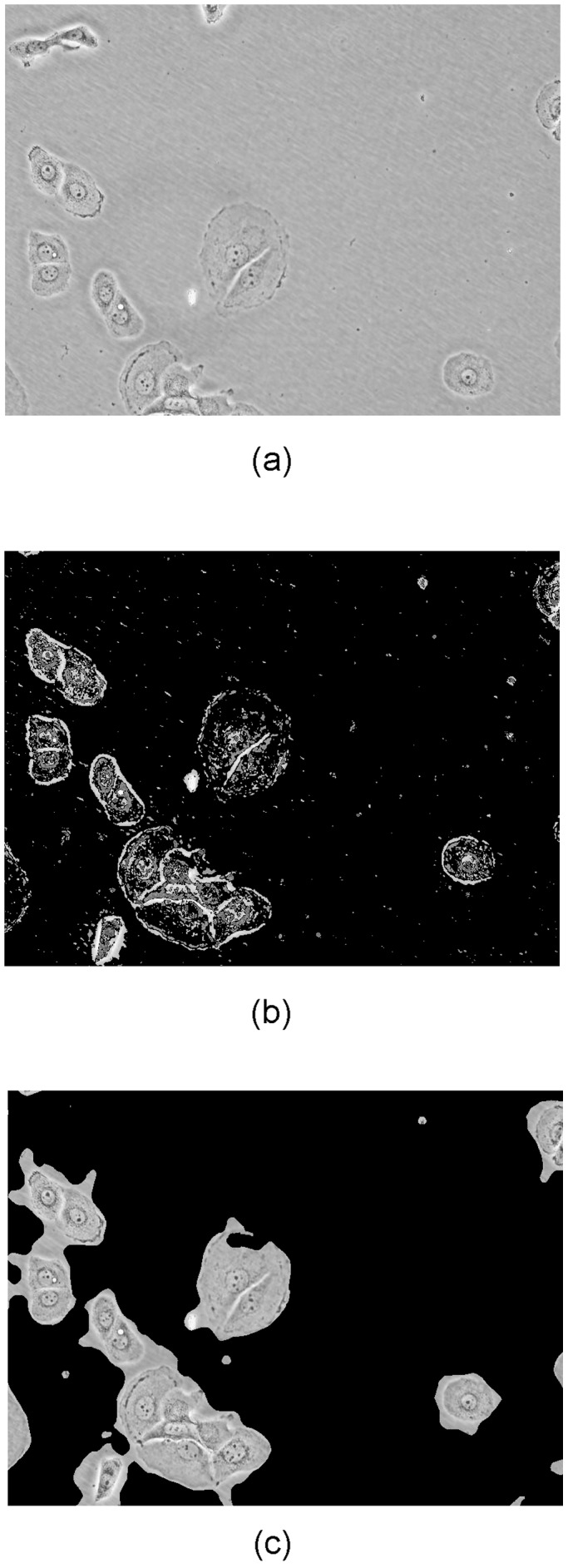
Filterbanks for the dual-tree complex wavelet transform.

The dual-tree complex wavelet transform (DT-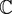
WT) has been recently used in various signal and image processing applications [18], [19], [20] and [21]. It has desirable properties such as shift invariance, directional selectivity and lack of aliasing. In the dual-tree 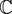
WT, two maximally decimated discrete wavelet transforms are executed in parallel, where the wavelet functions of two different trees form an approximate Hilbert transform pair [22]. Filterbanks for DT-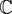
WT are shown in Figure 3. Low-pass analysis filters in real and imaginary trees must be offset by half-sample in order to have one wavelet basis as the approximate Hilbert transform of the other wavelet basis [23]. Analyticity allows one-dimensional DT-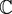
WT to be approximately shift-invariant and free of aliasing artifacts often encountered in DWT-based processing. Two-dimensional DT-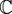
WT is also directionally selective in six different orientations, namely, 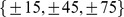. We acknowledge the fact that Gabor wavelets can also give derivative into different directions, but as pointed out in [24], “a typical Gabor image analysis is either expensive to compute, is noninvertible, or both. With the 2-D dual-tree CWT, many ideas and techniques from Gabor analysis can be leveraged into wavelet-based image processing”.

Microscopic cancer cell line images contain significant amount of oriented singularities. Recently, a Bayesian classification method that uses the sparsity in a transform domain is developed to classify cancer cell lines [25]. Attributes like orientation selectivity and shift invariance render DT-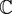
WT a good choice for the processing of microscopic images with lots of edge- or ridge-like singularities. We incorporate the complex wavelet transform into recently proposed region covariance descriptors [26] for feature extraction from microscopic images. In the region covariance framework each pixel is mapped to a set of pixel properties which's covariances are measured and used as a region descriptor. We use DT-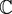
WT complex coefficient magnitudes in detail subbands as pixel features and compute covariance descriptors. Augmenting covariance matrices with directional information through the use of 2-D DT-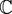
WT helps to improve the discriminative power of descriptors.

2-D DT-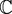
WT of an image is obtained by four real separable transforms [27]. Real-part and imaginary-part analysis filters are applied successively to rows and columns of the image. By addition and subtraction of corresponding detail subbands, we obtain a total of 16 subbands consisting of 6 real detail subbands, 6 imaginary detail subbands and 4 approximation subbands. Two-dimensional dual-tree decomposition is an oversampled transform with a redundancy factor of 4 (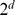 for d-dimensional signals). In our work, we perform two-level 2-D DT-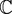
WT decomposition of each biomedical image of size 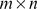 and use only the 2^nd^ level detail subband coefficients to better exploit the analyticity of DT-CWT. Each subband at the 2^nd^ level is of size 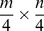. The original image is lowpass filtered with 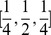 filters and downsampled by 4 in both directions to obtain a single intensity image 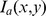 which represents the original image and will be used as the image to be classified. Let 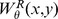 and 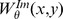 denote, respectively, the real and imaginary part of the 2nd level complex wavelet coefficient at the position (x,y) corresponding to directional detail subbands at orientation 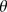, where 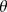 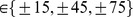. The magnitude of the complex wavelet coefficent is then given by
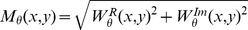
(1)


Hence, for each pixel in the average image 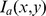, six complex wavelet coefficient magnitudes 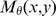 representing six different orientations of DT-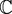
WT are extracted. These magnitudes will be utilized as features in the covariance matrix computation for randomly sampled regions of the image 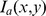. The computational complexity of (DT-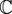
WT) is 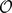 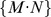, where 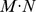 refers to the number of pixels in the image.

#### 2.2 Directional differences

In order to account for the large morphological variation of the images in our dataset, we evaluated differences between pixels in various directions. Consider a point 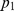 on a two-dimensional function 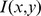. Now consider a second point 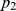. The Euclidean distance between 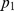 and 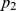 is 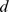 and 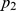 lies on line that has an orientation of angle 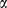 with respect to the 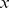-coordinate, i.e., 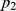 lies on a circle, which's center point is 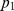 and has a radius 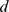. The difference between 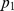 and 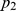 can be written as
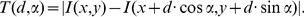
(2)


Now consider we want to compute a couple of difference values for equidistant concentric circles where the largest circle has radius 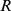 and the smallest has radius 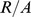, where 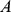 is an integer with values ranging from 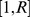. When the parameters 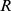 and 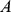 are fixed, we can rewrite the above equation as
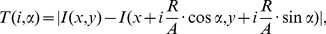
(3)where 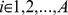. We can compute a score for each 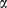 value by computing a function with respect to 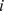, as


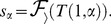
(4)

For example, 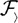 can be the median function. In that case 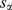 is simply the median of all the differences between the center pixel and the points at distances 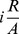 at the fixed orientation 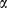. We use these scores as features in covariance matrix computation. Three different functions, namely median, max and mean functions, are employed for 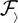 in this study. For each image 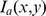 obtained according to the dual-tree complex wavelet section, 8 output images of the same size are generated as the result of the function 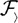, corresponding to 8 different orientations when the radius 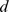 is chosen as 5 in the experiments. Hence, in addition to DT-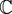
WT features, each pixel (x,y) of the image 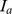 has 8 attributes, which denote the scores 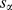 for 8 different 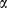 values.

The computational complexity of the directional difference operation is 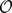 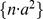, where 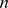 and 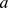 refer to the number of digits of the pixelsand the number of considered angles, respectively.

#### 2.3 Covariance matrices for cell line description

Successfully employed in texture classification [28], pedestrian detection [29] and flame detection [30], covariance descriptors enable the combination of different features over an image region of interest. Given an intensity image I of size 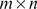, we define a mapping 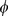 from image domain to feature domain as
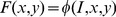
(5)where each pixel (x,y) is mapped to a set of features and F is the 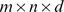 dimensional feature function. For a given subwindow R consisting of n pixels, let 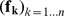 be the 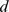-dimensional feature vectors extracted from R. Then, the covariance matrix of region R can be computed as
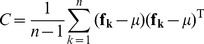
(6)where 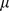 is the mean of the feature vectors inside the region R. The covariance matrix is symmetric positive-definite and of size *dxd*. There exists a very efficient multiplier-less implementation of covariance descriptors, called co-difference matrices, which have been shown to yield comparable performances to the original ones [31].

In this study, normalized covariance matrices are used as in [32].
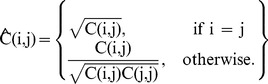
(7)With
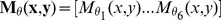
(8)and
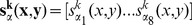
(9)where 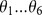 correspond to the six orientations of DT-CWT detail subbands 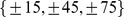, 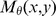 is as defined in Equation (1), 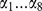 correspond to the eight orientations of directional difference score estimation and 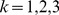 denote, respectively, the median, max and mean functions 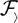 in the directional differences section, feature mapping functions employed in this study are



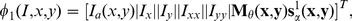
(10)

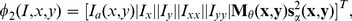
(11)

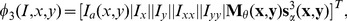
(12)

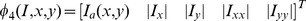
(13)where 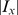 and 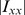 denote the first- and second-order derivatives at 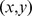 of the image 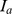.

The computational complexity of covariance matrix computation is 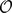 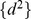, where 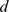 refers to the number of features in the subimage.

### 3 Classification Using a Multiclass SVM

**Figure 4:**
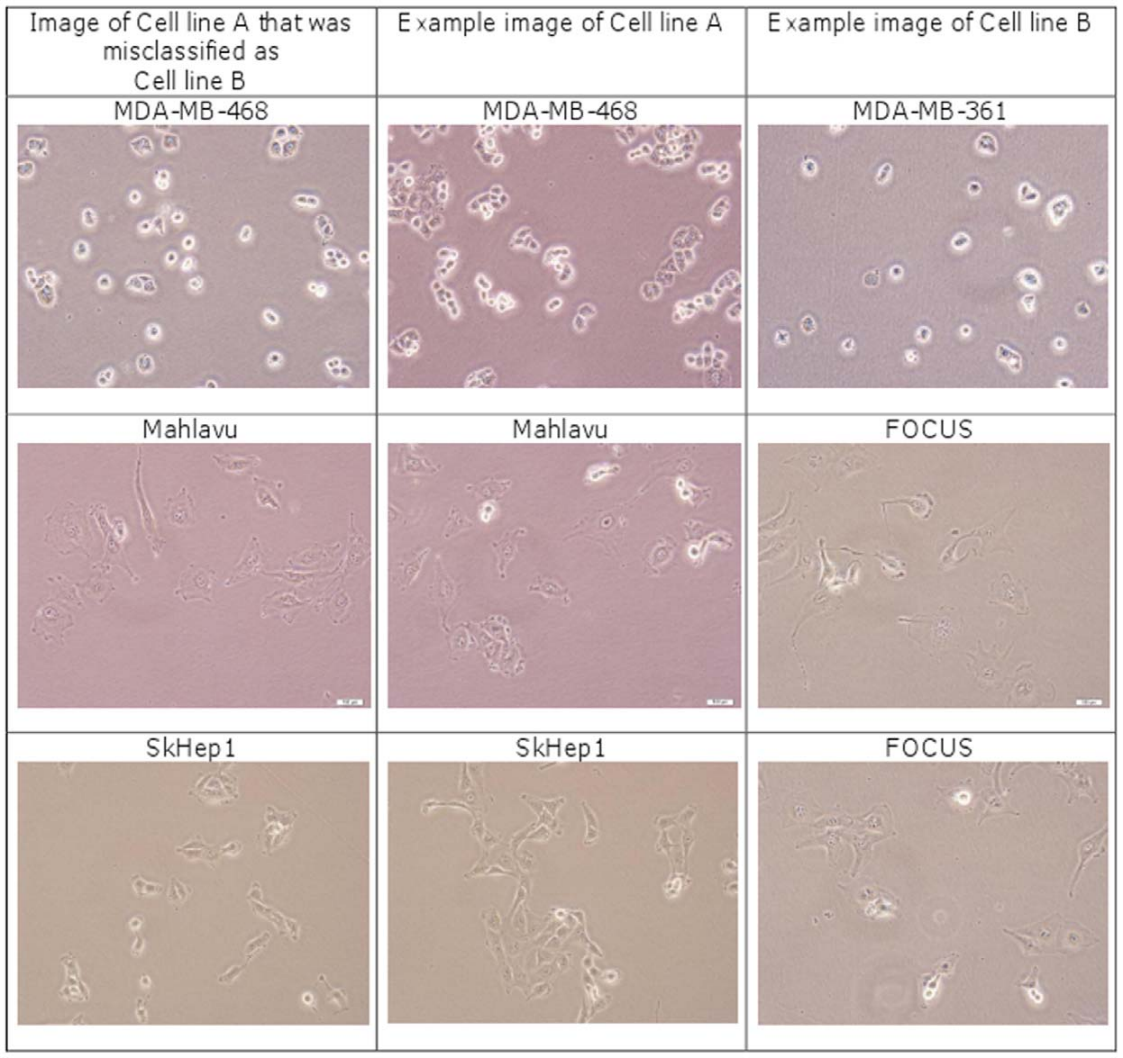
Examples of segmentation into foreground and background. a) Original image, b) EM Segmentation, c) EM segmentation followed by morphological closing and median filtering.

The images in our dataset show a large amount of background pixels. Clearly, the background is not discriminative. Therefore, we address the issue of segmenting the images into foreground and background before classification. For our dataset, a simple thresholding scheme is not sufficient for segmentation, since foreground pixels have a large variance and may therefore have values higher and lower than the background pixels. We modeled the image as a mixture of two Gaussians, representing the foreground and background pixels, respectively. Using this model, an Expectation-Maximization (EM) algorithm was applied for segmentation. The result is noisy, so a morphological closing operation was applied, followed by median filtering. We obtained the sizes of the closing and median filter kernels by comparing the scores of the segmentation results of various kernel sizes. The used score was first described in [33] and evaluated in [34]. Examples can be seen in Figure 4.

Since it is necessary to focus on foreground-like regions in carcinoma cell line images, 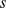 analysis square windows are randomly selected, as in [35], from each image with the two constraints: the percentage of the foreground pixels in the selected region of an image must be above 50 and the variance of the selected region must exceed an image-dependent threshold, which is the variance of the whole image.

For each subwindow, a covariance matrix is computed using Equation (6) for each of the feature mapping functions in (10)-(13). The image signature is composed of 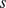 covariance matrices of the same size. Each class is represented by 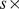
#(images in each class) covariance matrices. Covariance matrices are symmetric positive-definite and do not lie in the Euclidean space; so, they are vectorized resulting in 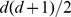-dimensional vectors for 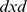 matrices. A multiclass SVM classifier is trained with RBF kernel in the 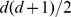-dimensional vector space using the training points. SVM algorithm is implemented using LIBSVM library [36]. For each test subwindow, the corresonding covariance descriptor is vectorized and fed into the trained SVM model for prediction. Therefore, there exist 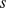 labels for each microscopic image corresponding to 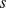 subwindows, and the image in question is assigned the label that gets the majority of votes among 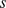 labels. The above process is re-executed using normalised covariance matrices instead of unnormalised covariance matrices. In order to compare the discriminative power of our features with more traditional one, we carried out similar experiments with SIFT [13] features for the 20× images. In SIFT, feature points are extremas in scale-space, i.e., a difference-of-gaussians (DoG) pyramid. The method is invariant to scale, orientation and location of the features, which makes it a commonly-used method in the field of computer vision. In our experiments, SIFT features are computed on the foreground that is found according to the description above. The resultant feature vectors for the images were then fed into an SVM. Table 6 shows the performance of those features. While the accuracy for discriminating between two cancer cell lines is 100%, the SVM classifier performs more poorly with each added cancer cell line.

The computational complexity of SVM classification in the test phase is 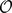 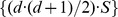 [37], where 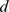 and 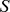 refer to the number of features and the number of support vectors, respectively.

## Availability and Future Directions

The software can be tested at http://signal.ee.bilkent.edu.tr/cancerCellLineClassificationEngine.html. The datasets used in this study can also be downloaded from there and can be used by fellow researchers in future studies. Images to be uploaded should be recorded using either 10×, 20× or 40× magnification and should be in *JPG* format. The authors are currently working on making the described procedure more computationally efficient by using a single-tree approximation to the dual-tree complex wavelet transform used in this study.

## Supporting Information

Data S1 **The supporting information consists of a RAR file named ‘Data S1.rar’.** This file includes several MATLAB files that can be used to evaluate the identity of test images provided by the user. Note that an online version of this program is available at http://signal.ee.bilkent.edu.tr/cancerCellLineClassificationEngine.html and a dataset of images is available at http://signal.ee.bilkent.edu.tr/cancerCellLineClassificationSampleImages.html.
(RAR)
